# The Role of Micro-RNAs and Circulating Tumor Markers as Predictors of Response to Neoadjuvant Therapy in Locally Advanced Rectal Cancer

**DOI:** 10.3390/ijms21197040

**Published:** 2020-09-24

**Authors:** Fatima Domenica Elisa De Palma, Gaetano Luglio, Francesca Paola Tropeano, Gianluca Pagano, Maria D’Armiento, Guido Kroemer, Maria Chiara Maiuri, Giovanni Domenico De Palma

**Affiliations:** 1 Equipe 11 labellisée Ligue contre le Cancer, Centre de Recherche des Cordeliers, INSERM UMRS 1138, Sorbonne Université, Université of Paris, 75005 Paris, France; kroemer@orange.fr (G.K.); chiara.maiuri@crc.jussieu.fr (M.C.M.); 2 Metabolomics and Cell Biology Platforms, Gustave Roussy Comprehensive Cancer Institute, 94800 Villejuif, France; 3 CEINGE-Biotecnologie Avanzate, 80131 Naples, Italy; 4 Department of Public Health, University of Naples “Federico II”, 80138 Naples, Italy; gaetano.luglio@gmail.com (G.L.); madarmie@unina.it (M.D.); 5 Department of Clinical Medicine and Surgery, University of Naples “Federico II”, 80138 Naples, Italy; fpt.tropeano@gmail.com (F.P.T.); gianluca.pagano94@gmail.com (G.P.); 6 Suzhou Institute for Systems Medicine, Chinese Academy of Sciences, Suzhou 100864, China; 7 Department of Women’s and Children’s Health, Karolinska Institutet, 171 77 Stockholm, Sweden; 8 Pôle de Biologie, Hôpital Européen Georges Pompidou, AP-HP, 75015 Paris, France; 9 Centro Interuniversitario di Studi per l’Innovazione Tecnologica in Chirurgia, University of Naples Federico II, 80138 Naples, Italy

**Keywords:** rectal cancer, locally advanced rectal cancer, LARC, neoadjuvant therapy, predictive biomarker, circulating biomarkers, microRNA, circulating miRNAs, circulating tumor cells, CTCs, cell-free DNA

## Abstract

The response to neoadjuvant chemoradiation (nCRT) is a critical step in the management of locally advanced rectal cancer (LARC) patients. Only a minority of LARC patients responds completely to neoadjuvant treatments, thus avoiding invasive radical surgical resection. Moreover, toxic side effects can adversely affect patients’ survival. The difficulty in separating in advances responder from non-responder patients affected by LARC highlights the need for valid biomarkers that guide clinical decision-making. In this context, microRNAs (miRNAs) seem to be promising candidates for predicting LARC prognosis and/or therapy response, particularly due to their stability, facile detection, and disease-specific expression in human tissues, blood, serum, or urine. Although a considerable number of studies involving potential miRNA predictors to nCRT have been conducted over the years, to date, the identification of the perfect miRNA signatures or single miRNA, as well as their use in the clinical practice, is still representing a challenge for the management of LARC patients. In this review, we will first introduce LARC and its difficult management. Then, we will trace the scientific history and the key obstacles for the identification of specific miRNAs that predict responsiveness to nCRT. There is a high potential to identify non-invasive biomarkers that circulate in the human bloodstream and that might indicate the LARC patients who benefit from the watch-and-wait approach. For this, we will critically evaluate recent advances dealing with cell-free nucleic acids including miRNAs and circulating tumor cells as prognostic or predictive biomarkers.

## 1. Introduction

Rectal cancer (RC) is one among the top four most deadly neoplasms worldwide [1]. In the last three decades, high-income countries experienced a reduction in the incidence of RC, as well as a decrease of its mortality, maybe due to both diagnostic/therapeutic improvements and secondary prevention. However, middle- and low-income countries have a constantly increasing incidence but lower if compared to the rest of the world.

Clinical presentation of RC includes a change in bowel habits (diarrhoea, constipation, frequents bowel movements), presence of haematochezia, rectal tenesmus, abdominal pain and systemic symptoms such as iron-deficiency anaemia, weight loss, and weakness.

The management of RC requires an effective coordination between healthcare professionals due to the interdisciplinary nature of its treatment pathway. The multidisciplinary team should be made of referral oncologists, colorectal surgeons, radiotherapists, radiologists, pathologists and endoscopists. The absence of any one of these figures may compromise the oncological outcome [2,3]. In fact, a retrospective analysis showed that unsuccessful multidisciplinary discussion was one of the predictive factors for positive resection margins, as well as the absence of radiotherapy [4].

In this review article, we will enumerate diagnostic and treatment challenges encountered when dealing with advanced rectal cancer. Reflecting huge advances in molecular biology, we will summarize the current knowledge of miRNAs as potential predictors of response to nCRT in LARC. Due to the increasing impact of circulating biomarkers in cancer care, we will also provide an overview of the involvement of circulating miRNAs, as well as single nucleotide polymorphisms (SNPs) associated with miRNAs (miR-SNPs) in the prediction of response to nCRT treatment. Finally, we will inspect the recent advances on the potential predictive role of other circulating tumor markers in LARC, with a particular focus on the cell-free (cf) nucleic acids and circulating tumor cells (CTCs).

## 2. Clinical Challenges in Rectal Cancer

### 2.1. Diagnostic Work-Up

Digital rectal exam represents the first and irreplaceable act to be performed in a patient with a suspect of RC: distance from the anal verge, tumor volume, location, presence of blood, and sphincter status are the parameters that can be evaluated. Colonoscopy is mandatory in order to confirm or disconfirm the clinical suspect of RC. More, the endoscopists can use confocal laser endomicroscopy (CLE) to assess the vascular microarchitecture in vivo and in a non-invasive way [5,6]. RC classification as low-middle-high RC (according to its distance from the anal verge, 5–10–15 cm, respectively) and intraperitoneal/extraperitoneal is crucial for further management steps. Intraperitoneal RC can be treated as colon cancer while extraperitoneal RC requires different staging and therapeutic procedures. In fact, in early stages (tumor stage 1–2 (cT1–cT2), when cancer only invades the submucosa or muscolaris propria, respectively), local staging is obtained by means of transrectal ultrasonography (TRUS), while pelvic Magnetic Resonance Imaging (MRI) is the first-choice imaging technique for locally-advanced cancers [7]. MRI plays a crucial role in identifying different risk groups among patients with rectal cancer and helps selecting the most appropriate neoadjuvant therapy according to the risk of local recurrence and distant metastasis.

Computed Tomography (CT) of the chest and the abdomen is preferred to assess the presence of metastasis, thus completing the staging.

### 2.2. Locally Advanced Rectal Cancer (LARC)

When a rectal cancer is diagnosed as locally advanced (cT3-T4, invading the perirectal fat or adjacent structures, respectively), or with metastatic perirectal lymph-nodes (N+), multimodal strategies with perioperative radiochemotherapies become crucial to optimize the outcomes.

The combination of neoadjuvant chemoradiotherapy (nCRT) and total mesorectal excision (TME) has become the standard of care for LARC, highlighting the importance of multidisciplinary team work-up [8]. TME is still a milestone in the treatment of RC: first described by Heald et al., this surgical procedure can be performed via open, laparoscopic, and transanal surgery (TaTME) [9,10]. It consists of the complete removal of the perirectal tissues involving lateral and circumferential margins of the rectal envelope as well as the organ itself.

To investigate the efficacy of short-term preoperative radiotherapy combined with TME, the Dutch Colorectal Cancer Group launched the “TME trial”, a multicenter randomized controlled trial in which 1805 eligible patients with RC were randomly assigned to one of the two study branches: either preoperative 5 Gy radiotherapy (RT) plus TME (897 patients) or TME alone (908 patients) [11]. Two-year follow-up results demonstrated that the rates of local recurrence were 2.4% in the RT + TME group and 8.2% in the TME alone one (*p* < 0.001). According to the univariate analysis, the hazard ratio (HR) for local recurrence in the group assigned to TME alone, compared with the group assigned to preoperative RT + TME was 3.42 with a 95% confidence interval (CI) of 2.05 to 5.71. Moreover, the overall recurrence rate was 16.1% in the RT + TME group and 20.9% in the group assigned to TME alone (*p* = 0.09). The HR for any recurrence in the TME-only group as compared to the RT + TME group was 1.21 with a 95% CI of 0.97 to 1.52. After a median follow-up of six years, the authors reported that the five-year local recurrence risk of patients undergoing a macroscopically complete local resection was 5.6% if preoperative radiotherapy was conducted compared with 10.9% in patients undergoing TME alone (*p* < 0.001). Five-year overall survival (OS) rates were 64.2% and 63.5%, respectively (*p* = 0.902). Subgroup analyses revealed a significant effect of radiotherapy in reducing local recurrence risk for patients with nodal involvement, for patients with lesions between 5 and 10 cm from the anal verge, and for patients with uninvolved circumferential resection margins [12].

After a median follow-up of 12 years, preoperative short-term radiotherapy for patients with resectable rectal cancer decreases local recurrence rates by more than 50% in comparison to the surgery-alone strategy, with a decreased overall recurrence rate. In fact, the 10-year cumulative incidence of local recurrence was 5% in the group assigned to radiotherapy and surgery and 11% in the TME-alone group (*p* < 0.0001). Overall survival did not differ between groups. For patients with stage III cancer, according to the tumor, node, and metastasis (TNM) system, with a negative circumferential resection margin, 10-year survival was 50% in the preoperative radiotherapy group versus 40% in the surgery-alone group (*p* = 0.032) [13].

### 2.3. The Concept of Tumor Regression Rate

Once nCRT is completed, it is useful to assess the tumor regression grade (TRG), which is characterized by a strong prognostic value [14,15]. So far, many TRG scales have been proposed in the literature and the most widely used are the Mandard scale, the Dworak scale and the Ryan scale (Table 1) [16,17,18].

Mandard and colleagues proposed a TRG system for esophageal carcinoma, which has been adopted in other digestive tract malignancies (Table 1). TRG was quantitated in five grades: 1 (complete regression) showed absence of residual cancer and fibrosis extending through the different layers of the esophageal wall; 2 was characterized by the presence of rare residual cancer cells scattered through the fibrosis; 3 was characterized by an increase in the number of residual cancer cells, but fibrosis still predominated; 4 showed residual cancer outgrowing fibrosis; and TRG 5 was characterized by absence of regressive changes (Table 1).

According to Dworak and collaborators., TRG can be classified in four grades: TRG 0, with no regression; TRG 1 represents minimal response, with dominant tumor mass, obvious fibrosis and vasculopathy; TRG 2 is characterized by a moderate response with dominant fibrotic changes and a few easy-to-find tumor cells or groups; TRG 3 indicates a near-complete response with few microscopically difficult-to-find tumor cells in fibrotic tissue with or without mucous substance; TRG 4 defines a complete response with no tumor cells and only fibrotic mass or acellular mucin pools (Table 1).

**Figure 1:**
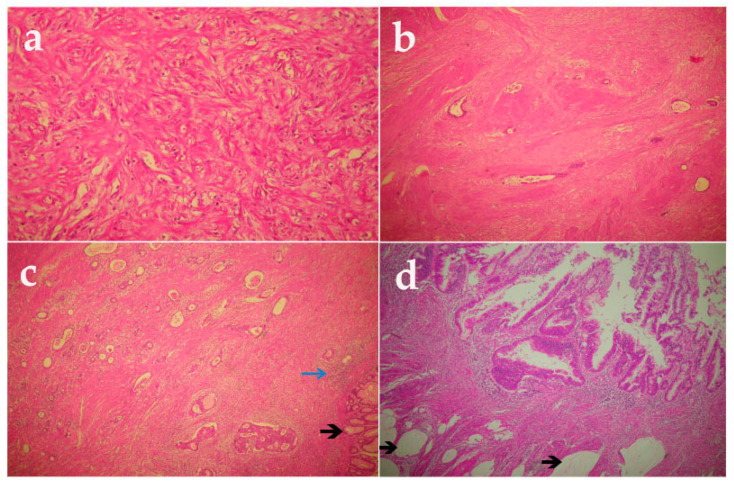
Representative histological appearance of modified Ryan TRG scale. (**a**) Complete tumor regression (TRG 0) (20×); (**b**) Near-complete response (TRG 1) (10×); (**c**) Partial response (TRG 2). Areas of fibrosis and focal flogosis (blue arrow), and neoplastic glands (black arrow) (10×) are shown; (**d**) Poor o no response (TRG 3). Black arrows indicate acellular mucin pools in the context of neoadjuvant therapy (20×). (**a**–**d**) Hematoxylin and eosin staining sections. TRG, tumor regression grade.

Yet another classification proposed by Ryan et al. is currently advised by the American joint committee on cancer (AJCC) Cancer Staging Manual VIII ed. [18,19]. In this classification, the tumor regression score varies from 0, indicating complete response, to 3 referring to poor or no response (Table 1). In Figure 1, pathologic images of different types of tumor regression grades according to the Ryan classification are illustrated, showing the balance between residual tumor and post-radiotherapy fibrosis.

Interestingly, data from literature show a wide variety of response to neoadjuvant treatments, with complete clinical responses ranging from 8 to 30% [20,21,22,23,24,25,26].

This finding is someway intriguing, considering that we are facing rectal cancers, which, at the beginning, are similarly staged as locally advanced. Bearing this in mind, one might wonder: why similar radiochemotherapies protocols led to such different results?

Slight differences in chemo or radiation doses may only partially explain these results; cancer-related and biological features are, on the other hand, certainly implicated in the variability of the response.

### 2.4. Assessing Clinical Response

Considering the lack of standardization in assessing the complete clinical response (cCR), Habr-Gama et al. provided an attempt to guide healthcare professionals to its recognition [27]. Whitening of the mucosa in an area of the rectal wall, the presence of any telangiectasia, loss of pliability of the rectal wall harboring the scar and the absence of the tumor were marked as endoscopic features of complete clinical response. On the other hand, incomplete clinical response should be suspected when residual deep or superficial ulcerations, palpable nodules, or any significant stenosis, which does not allow the proctoscope to slide through, are found.

Endoscopic aspects associated with cCR were not the only ones to be evaluated so far. In fact, Lambregts et al. ran an observational monocentric study in which they described the MRI morphology of the rectal wall in patients who gained cCR after nCRT for LARC [28]. The Authors enrolled 19 patients who underwent nCRT and watch-and-wait management strategy with both short-term (≤6 months) and long-term follow-up (>6 months). Short-term follow-up revealed four different MRI patterns: minimal fibrosis causing slight local thickening of the rectal wall was observed in 7/19 patients; full-thickness fibrosis was detected in 3/19 patients who had bulky tumor types, in whom the previous isointense tumor became fully hypointense; 4/19 patients developed irregular fibrosis; 5/19 patients showed a completely normal 2-layered rectal wall. These findings were confirmed at long-term follow up: the aspect of rectal fibrosis tended to remain stable with time and, for those patients who showed a completely normal rectal wall, there was no evolution into fibrosis [28].

Positron emission tomography/computed tomography (PET/CT) features in assessing complete clinical response were investigated by Perez et al. [29]. In their study, 99 patients were included and underwent baseline PET/CT before nCRT and two additional PET/CT at 6 weeks and at 12 weeks after radiotherapy completion. Ninety-one underwent 6-week PET/CT, and 99 underwent 12-week PET/CT imaging. Standard Uptake Value (SUV) was advocated as predictor of response. In fact, both mean SUVmax at 6 weeks and mean SUVmax at 12 weeks were significantly lower among patients with complete response to CRT [3.6 vs. 6.8; (*p* = 0.001) and 2.8 vs. 8.3; (*p* < 0.0001) respectively]. In addition, the variation between 12-week SUVmax and baseline PET/CT was also significantly higher in patients with complete response (80 vs. 50%; *p* < 0.0001) [29].

### 2.5. The Watch and Wait Approach

Fully considered as one of organ-preserving strategies, this approach was advocated by Habr-Gama and colleagues in several studies [30,31,32,33]. Their single-center prospective study showed that an alternative nCRT regimen and a non-operative management may result in high rates of sustained complete clinical response [34]. Precisely, patients received 54 Gy of radiation and 6 cycles of chemotherapy: 45 Gy of radiation were delivered with 1.8 Gy daily doses to the pelvis followed by additional 9 Gy to the primary tumor and perirectal tissue; at the same time, they administered 3 cycles of 5-Fluorouracil (5-FU) (450 mg/m^2^) and 50 mg of leucovorin for 3 consecutive days every 3 weeks. Once the radiation is completed, patients received 3 identical cycles of chemotherapy every 3 weeks. As for early tumor regrowth, complete clinical response at 10 weeks from RT completion was achieved in 47 patients (68%) with a three-year OS and disease-free survival (DFS) of 90% and 72%, respectively. The complete clinical response was sustained by 39 patients for at least 12 months of follow-up and 4 of them had a late local recurrence between 13 and 35 months from nCRT completion. The remaining 35 patients (51%) benefited of a complete clinical response, which never required surgery after a median follow-up of 56 months with a three-year overall and disease-free survival of 94% and 75%, respectively [34].

Nevertheless, given the lack of randomized controlled trials (RCT), the watch and wait approach is a highly debated strategy [35,36]. To date, in addition to small/moderate-sized series from referral centers, a consistent part of evidence arises from the International Watch & Wait Database (IWWD): it aims to describe the outcome of the watch and wait strategy in a fifteen-country-wide registry of pooled individual patient data [37]. This is the largest series on this topic, including 880 patients with a complete clinical response, and it reports a local regrowth rate of 25.2%, especially in the first 2 years of follow-up, with a 5-year OS and 5-year disease-specific survival of 85% and 94%, respectively [37].

More, a propensity score-matched cohort analysis study (the oncological outcomes after clinical complete response in patients with RC (OnCoRe) project) included patients of all ages diagnosed with rectal adenocarcinoma without distant metastases who had received preoperative chemoradiotherapy at a tertiary cancer center between 2011 and 2013 [38]. Patients who had a clinical complete response were offered the watch-and-wait approach, and patients who did not have a complete clinical response were managed with surgical resection if eligible. The primary endpoint was non-regrowth disease-free survival from the date that chemoradiotherapy was started, and secondary endpoints were overall survival and colostomy-free survival [38].

After a median follow-up of 33 months, 44 of the 129 patients managed by watch and wait (34%) had local regrowth; 36 (88%) of 41 patients with non-metastatic local regrowth were salvaged. In the matched analyses (109 patients in each treatment group), no differences in 3-year non-regrowth disease-free survival were noted between watch and wait and surgical resection (88% with watch and wait vs. 78% with surgical resection). 3-year overall survival was similar for both groups (96% vs. 87%). Patients managed by a watch-and-wait strategy had better 3-year colostomy-free survival than did those who underwent immediate surgical resection (74% (95% CI 64–82) vs. 47% (37–57)) [38].

Further information might be highlighted by a randomized phase II trial, which is still in progress (NCT02052921), comparing oncologic outcomes between surgical and observational treatment in patients who underwent nCRT for locally advanced rectal cancer with complete clinical response.

### 2.6. Factors Associated with Response to Neoadjuvant Chemoradiation in LARC Patients

About 20% of patients with a clinical complete response after neoadjuvant chemoradiation, will develop early tumor regrowth. With different studies, Habr-Gama and collaborators tried to identify possible factors related to the response to neoadjuvant chemoradiotherapy: one of these factors was baseline radiological t classification [39]. In a retrospective review of a prospective collected database, Habr-Gama et al. included 91 patients with distal rectal cancer (≤7 cm from the anal verge) cT3 or N+ cancers and those with ultralow cT2N0 (only invading the muscolaris propria without metastatic perirectal lymph-nodes), and candidates to neoadjuvant chemoradiotherapy (with 54 Gy and bolus 5-fluorouracil and leucovorin delivered in 6 cycles, 3 concomitant to radiation and the remaining 3 after radiation completion) [39]. At 10 weeks from the last day of radiotherapy completion, a digital rectal examination (DRe) and rigid proctoscopy were performed by colorectal surgeons to assess the response of the tumor to the therapy, and patients with presence of a normal DRe or only subtle induration, presence of whitening of the mucosa, telangiectasia without any ulceration, mass, or stenosis of the rectum, were classified as “patients with clinical complete response and candidate for watch-and-wait approach.

Sixty-seven % of patients developed a cCR at the first assessment after 10 weeks from radiation completion and were offered the option to watch and wait. Among these, 29% (18 patients) developed local recurrences (presence of adenocarcinoma within the rectal wall, mesorectum, or pelvis), of whom, 18% (11 patients) were classified as patients with “early tumor regrowths” (within the first 12 months of follow-up); on the other hand 11% (7 patients) of patients developed it after the 12 months and classified as “late tumor regrowths”. A univariate analysis was performed with the aim to identify potential risk factors related to the development of early tumor regrowth: the only statistically significant related factor was the baseline tumor (T) classification (T2 vs. T3/T4). cT2 and cT3/T4 tumors developed similar initial cCR (72% vs. 63%; *p* = 0.40); out of 18 patients with local recurrences, 13 patients were baseline T3/T4 tumors (of whom 77% with early tumor regrowths, and 23% late tumor growths) and 5 were baseline cT2 tumors (of whom 20% with early tumor regrowths, and 80% late tumor growths). Local recurrence-free survival was significantly better for cT2 patients at 1 year (96% vs. 69%; *p* = 0.009) [39].

Overall survival for patients not developing local recurrence was 93.7%, whereas for patients with local recurrence and salvage resection, it was 63.3% at 5 years (*p* = 0.03). Thus, ct2 tumors are less likely to develop local recurrence, and patients with cT3/4 should be considered patients at high risk of developing local recurrence and this could be an important aspect to take into account when tailoring treatment and follow-up strategies.

Another important aspect to consider during the neoadjuvant regimen treatment is related to the fact that there are specific areas of the tumor more affected than others, and that there are areas more resistant to the radiotherapy treatment, and others more sensitive. Chand et al. proposed a personalized treatment strategy in order to reduce the toxicity of radiotherapy, modify doses and focus its action on more sensitive and more specific areas: the use of MRI linac (linear accelerator), which combines a modified 1.5-tesla(T) MRI (to prevent magnetic interference) and a 6-megavolt (MV) radiotherapy linear accelerator that allows to acquire real time images related to the response of the tumor to the radiotherapy treatment [40]. Based on the obtained acquisitions, this could allow to modify the radiotherapy doses, evaluate possible dose escalation, select more specific areas to focus the treatment and saving fibrotic tissue areas (complete tumor regression sensitive).

To date, clinical and radiological assessment of tumor response to nCRT does not allow the accurate identification of patients with complete pathological response (pCR). Therefore, the identification of many types of biomarkers, such as microRNAs (miRNAs) and circulating tumor cells able to predict responsiveness to nCRT, may support and improve the clinical decision-making in identifying which patients could benefit from the watch-and-wait approach.

## 3. microRNAs in Locally Advanced Rectal Cancer

### 3.1. microRNAs as Potential Biomarkers in LARC: The beginning of the Investigation

As described before, in the management of LARC patients, the identification of valid biomarkers for choosing the best clinical decision-making approach could help in separating in advance responders from non-responders to nCRT. In this context, miRNAs seem to be promising candidates in predicting LARC prognosis and/or therapy response, particularly due to their stability, easily detection and disease-specific expression in human tissues, blood, serum, urine and other non-invasive sources [41,42].

miRNAs are a family of small (about 22 nucleotides in length), single stranded and non-protein encoding RNAs [41]. They exert their main function at the post-transcriptional level, especially by inhibiting the expression of target genes [41,42]. This mechanism of gene silencing is mainly exploited by the direct binding of miRNAs to complementary sequences on the 3′-untranslated regions (UTRs) of messenger RNA transcripts (mRNAs) that consequently induces mRNA degradation or translational repression; moreover, it could be indirectly influenced by epigenetic mechanisms [43]. Additionally, several findings reported that the binding of miRNAs to other regions of target genes (i.e., protein-coding exons) could induce gene expression [44].

The key regulatory role of miRNAs translates in their involvement in a variety of biological processes (i.e., cell proliferation and differentiation, apoptosis and development) [41]. Over the past decade, it has been widely demonstrated that miRNAs are deregulated in a variety of tumors [41]. The altered levels of expression of miRNAs could be caused by multiple mechanisms, such as epigenetic alterations, chromosomal abnormalities, as well as transcriptional control changes [45]. Depending on the target gene, miRNAs are recognized as having an oncosuppressive or oncogenic (oncomiR) role in the pathogenesis and/or progression of human cancers, including LARC [41,46,47]. In particular, the increased expression of miRNAs mediates the silencing of tumor suppressor genes. Conversely, the reduced expression of miRNAs results in the overexpression of target oncogenes.

Normal and tumor samples present a unique signature of expression of miRNAs, as demonstrated by different genome-wide profiling studies [48,49]. Therefore, the assessment of the levels of expression of a single specific miRNA or signature of miRNAs in cancer specimens is a potential help in the clinical practice to predict with high accuracy the disease status, different types of tumors, and even the origin of tumors. Moreover, the usefulness of miRNAs is not only limited to diagnostic purposes, but it is also extended to prognostic and predicting values [50]. In this scenario, a variety of miRNAs have been identified as deregulated in colorectal cancer (CRC) and the majority of them derive from studies performed on cell lines [47].

Concerning LARC, early-published studies had modest sample sizes and relied particularly on the identification of unique miRNA patterns that were able to distinguish between colon and rectal cancers, being them clinically and molecularly different. Moreover, the assessment of signatures of miRNAs was mainly based only on cross-validation. Accordingly, Slattery and colleagues evaluated differences in the expression of miRNAs by comparing a total of seventy colon and rectal tissues with specific tumor alterations (i.e., C phosphate G (CpG) island methylator phenotype positive (CIMP+), microsatellite instability positive (MSI+), Kirsten rat sarcoma viral oncogene (KRAS2)-mutated and tumor protein 53 (TP53)-mutated and 30 normal tissues obtained from paraffin-embedded tissues, by using a microarray approach [51]. The miRNA signatures identified, being specific of each type of tumor, were able to separate colon and rectal cancers [51]. However, the information obtained were limited to the tumor characteristics (i.e., CIMP). Additionally, the levels of miRNAs expression were not quantitatively validated by other methods, such as realtime PCR (RT-qPCR).

The rectal cancer miRNAome without any limitation to tumor molecular features was fully explored in 57 LARC tissues and the corresponding matched normal mucosa biopsies [52]. A total of forty-nine miRNAs differentially expressed (DE) between normal and rectal tissues were identified through Locked Nucleic Acid (LNA)-enhanced miRCURY^TM^ microarrays [52]. Among all the miRNAs detected, eight out of the 10 miRNAs were validated by semi-quantitative RT-qPCR and their levels of expression matched with the microarray data. Moreover, a signature of 14 miRNAs, specifically associated with rectal cancer, was discovered (miR-492, miR-542-5p, miR-584, miR-483-5p, miR-144, miR-2110, miR-652, miR-375, miR-147b, miR-148a, miR-190, miR-26a/b, and miR-338-3) by combining the miRNA profile obtained from LARC tissues with in silico data on miRNAs associated only with colon cancer [52].

### 3.2. microRNAs as Predictive Tissue Biomarkers in LARC

Over time and in line with the clinical advances, more and more studies have attempted to profile a combination of aberrant miRNAs in the complete pathological response to nCRT in LARC patients. Nevertheless, there are significant discrepancies among reported signatures of miRNAs. In Supplementary Table S1 are listed the most significantly miRNAs that are predictive of responsiveness to nCRT.

At the beginning, the most significantly deregulated miRNAs that were already known in the literature to play a role in CRC were also examined in LARC biopsies. Changes in the levels of expression of miR-125b and miR-137 in 31 patients affected by rectal adenocarcinoma were associated with worse tumor response to chemotherapy [53]. miR-125 and particularly miR-137, measured through RT-qPCR, were overexpressed after two weeks of capecitabine-based treatment. However, in the evaluation of the relationship between the tumor regression and miRNAs expression, only tumors with a weak or absent response to the treatment (TRG 3–5) showed both miRNAs as significantly upregulated (miR-125b fold change = 2, *p* = 0.023; miR-137 fold change = 6.3, *p* = 0.002) [53]. On the other hand, responder patients (TRG 1–2) did not reveal significant changes in miRNAs expression (miR-125b fold change = 2.3, *p* = 0.09; miR-137 fold change = 2.9, *p* = 0.058) [53]. Although the different comparisons made in this latter article to potentially define miR-125b and miR-137 as predictive biomarkers, the small number of patients, the inter-tumoral variability and the absence of a validation in a larger cohort limited their clinical applicability.

The deregulated expression in CRC of the oncomiR-21, along with the tumor suppressor miR-143 and miR-145 has been explored in pre- and post-therapeutic formalin-fixed, paraffin-embedded (FFPE) tumor tissues of LARC patients subjected to nCRT (5-FU and 50.5 Gy) [54]. The expression of miR-21 was higher in tumor tissues that in non-tumor specimens. However, differently from miR-143 and miR-145, miR-21 was found significantly downregulated also in tumor tissues collected after the nCRT therapy when compared to pre-therapeutic tumor tissues [54]. The high post-therapeutic expression of miR-143 and -145 did not correlate with a better overall survival. Additionally, by associating the levels of expression of miR-145 with the tumor regression grade, the upregulation of miR-145 positively correlates only with the TRG 2 and 3. In contrast, curiously, LARC patients with a complete response to the neoadjuvant therapy (TRG 4) and not responder patients (TRG 1) presented a marked low expression of miR-145 [54]. These latter results clearly confuse in asserting miR-145 as a predictive biomarker.

Subsequently, higher levels of miR-145 were identified in LARC patients who responded well to treatment (TRG 3–4) when compared with patients who demonstrated a poor response to therapy (TRG 1–2) [55].

Scarpati and colleagues identified a set of 13 miRNAs associated with a complete pathological response to neoadjuvant chemoradiotherapy (capecitabine and oxaliplatin in combination with one dose of 45 Gy) in 38 frozen tumor biopsies through a microarray approach and validated in their deregulated expression by RT-qPCR [56]. In particular, two miRNAs (miR-720 and miR-1274b) were downregulated, while 11 (miR-1183, miR-483-5p, miR-622, miR-125a-3p, miR-1224-5p, miR-188-5p, miR-1471, miR-671-5p, miR-1909*, miR-630, miR-765) were upregulated in patients that completely respond to the therapies (TRG 1) when compared to non-responders (TRG > 1, according to Mandard’s scale). Among them, miR-630 and miR-622 reached the 100% sensitivity and specificity in selecting TRG 1 cases, being both upregulated in all responders and downregulated in all non-responder patients [56]. Differently from the cited studies so far, this latter extended its research also to the prediction of the targets of the 13 miRNAs in order to clarify their role in response to the treatment. Indeed, proteins involved in the mechanisms of DNA repair and downstream to the epidermal growth factor receptor (EGFR), as well as poly (ADP-ribose) polymerase 3 (PARP3) and the insulin-like growth factor receptor (IGFR) pathways were revealed through a bioinformatics approach [56]. However, it still remains the need of a further exploration of the identified targets by functional wet-lab experiments, as well as the validation of the expression of at least miR-630 and miR-622 in an extended cohort of responder/non responder samples.

Years thereafter, miR-622 was reported highly expressed in pretreatment CRC cells of TRG 4 patients and post-treatment radiation-resistance cells with respect to pretreatment CRC cells of TRG 1–3 patients and post-treatment radiation-sensitive CRC cells [57]. Moreover, the overexpression of miR-622 was also reported in tumor specimens of non-responder LARC patients (TRG 4, according to Mandar’s scale) who were subjected to nCRT therapy (capecitabine plus oxaliplatin in combination with 45Gy of pelvic conformal radiotherapy) [57].

A signature of 8 differentially expressed miRNAs was linked to the positive tumor response to nCRT treatment (capecitabine or 5-FU with 45 Gy to pelvis plus 5.6 Gy boost to tumor) [58]. Particularly, miR-215, miR-190b and miR-29b-2* showed low expression levels, while let-7e, miR-196b, miR-450a, miR-450b-5p and miR-99a* were overexpressed in responder patients (TRG1–3, according to Mandard’s stage) [58]. Of these, none of the miRNAs matched with the previously cited ones. Different types of therapies and doses, as well as the different detection methods could in part explain this discrepancy. The study is limited due to the small number of LARC patients (*n* = 20), the disadvantages of being retrospective and the absence of validation of miRNAs in larger dataset with alternative techniques. Moreover, given the correlation between better prognosis and the complete response to preoperative CRT, it would be interesting having information on the interplay between the modulated expression of the detected miRNAs and the survival of responder patients.

Similarly, another expression signature composed of five miRNAs that was predictive of response to nCRT was discovered in 12 FFPE pre-treatment LARC tissue samples [59]. In particular, three miRNA transcripts (miR-16, miR-590-5p and miR-153) were able to distinguish between a complete and an incomplete nCRT response, and two miRNA transcripts (miR-519c-3p and miR-561) to predict good versus poor response with a median accuracy of 100%. However, the levels of deregulation of the cited miRNAs, in terms of down or over-expression, were not reported [59].

In modest sizes of frozen biopsies (*n* = 22), Hotchi and colleagues proposed miR-223 as a novel predictive biomarker, being significantly upregulated in responder LARC patients (*n* = 17; 40 Gy plus S-1) when compared to non-responders (*n* = 7) [60]. Furthermore, its expression has been validated in a separate cohort of samples (*n* = 21). Interestingly, miR-223 presented high values of expression in responder LARC patients with regard to histopathological examination and response evaluation criteria in solid tumors (RECIST), as well as downstaging [60].

Due to the incongruences among the aforementioned investigations, later studies started to evaluate larger cohorts of samples, as well as to include independent patient cohorts in order to validate the identified signatures and also to focus on the intimate wet-lab evaluation of miRNA functions and interplays. Moreover, one study explored changes in the expression patterns of miRNAs as a direct consequence of DNA copy number variations (CNVs) [61]. CNVs reflect the genomic imbalance of cancer [62]. They consist in structural variants that include insertions, deletions and amplifications of segments of DNA in the human genome, usually of 1 kilobase (kb) or larger [63]. DNA copy number of the miR-17-92a-1 cluster host gene (MIR17HG) has been determined in 108 tissues biopsies of individuals affected from LARC [61]. Indeed, the amplification of the MIR17HG gene was related to a lack of nCRT response. However, no significant association with response to treatment was detected when the expression levels of each single MIR17HG member (miR-17, miR-18a, miR-19a, miR-19b-1, miR-20a, and miR-92a-1) were evaluated by RT- qPCR [61].

Using miRNA microarray techniques, a total of 22 miRNAs were found as significantly upregulated in LARC patients who completely or partially responded to nCRT when compared to non-responders [64]. In particular, six miRNAs (miR-19-3p, miR-866-3p, miR-923, miR-494, miR-513a-5p and miR-513b) were elevated in responders who received tegafur/gimeracil/oteracil CRT, and 16 miRNAs (miR-154, miR-379, miR-223, miR-1542-5p, miR-144, miR-363, miR-31, miR-1290, miR-382, miR-193a-5p, miR-451, miR-335, miR-486-5p, miR-1246, miR-34b* and miR-144*) in responders treated with tegafur-uracil combined with a total of 40 Gy radiations [64].

The implication of the well-known oncomiR-21 also in prediction of response to preoperative CRT has been confirmed in seventy FFPE tumor tissues of LARC patients who underwent to CRT thanks to the work of Caramés et al. [65]. Subsequently, the same authors identified another predictive miRNA biomarker, namely miR-31 [66]. Differently from a previously published study, in this work miR-31 overexpression correlated with an unfavorable response to nCRT and OS in LARC patients [64,66].

In a study cohort of 45 LARC patients, a total of 12 miRNA, of which 11 downregulated (miR-30b, miR-145, miR-148a, miR-375, miR-451, miR-519b-3p, miR-650, miR-1183, miR-1233, miR-1243, and let-7f) while 1 upregulated (miR-18a), have been identified in responders (TRG 1 and 2, according to Mandard’s TRG scale) when compared to non-responder individuals [67]. Notably, a significant negative correlation was found between miRNA-375 expression and c-Myc expression [67]. Earlier investigations described the altered expression of some of the cited miRNAs, including miR-145, -451 and -1183 in LARC responder patients [54,56,64]. However, these different studies did not report the same levels of miRNAs deregulation.

Campayo and collaborators also identified miR-375, together with miR-21 and miR-99 as miRNAs related to nCRT response [68]. In particular, receiver operating characteristic (ROC) curve analyses evidenced the potential clinical utility of the combination of low levels of expression of these three miRNAs to distinguish patients with complete response (TRG 4) from patients without response or minor response (TRG 0–1) to nCRT [68].

Several of the miRNAs already identified and cited so far, including miR-21, -31, -125b, -145 and -630, were validated in a subsequent study of Eriksen and collaborators by RT-qPCR [69]. This latter investigation included a test (*n* = 55) and a validation cohort (*n* = 130) of LARC tissue specimens. High expression levels of miR-21 epitomized a major response to CRT and were associated with TRG 1 and TRG 2 tumors. Furthermore, LARC patients bearing low expression of miR-125 and miR-145 had an increased DFS [69]. However, the obtained results did not overlap in both cohorts analyzed in the cited work. Additionally, they were only partially in accordance with the data of the previous published studies. These diverging results could be ascribed to several factors, including the differences in the tumor content of sample specimens, the study cohorts, chemotherapy regimen, classification of responders, and platforms, as well as the tools used to analyze the miRNA-signatures. This clearly provides evidence that the necessity of validation of the miRNAs identified as putative predictive biomarkers.

It is also important to note that miRNAs belong to a highly complex system of regulation. Their altered expression is, in fact, only one of the multiple events that trigger or promote the malignant transformation and in the interplay between tumor tissues and the surrounding stromal tissues. The identification of specific miRNAs in responder patients based only on the measurement of the changes in their expression, without any functional inspection and, particularly, without their validation by means of non-invasive specimens, may compromise their clinical applicability. Moreover, most of the published literature reports information on the implication of the miRNAs on the local response to therapy, but not on prognosis. Only recently, it has been demonstrated that responder patients to the preoperative CRT had a better prognosis, with lower rates of recurrence and metastasis [70].

In this context, a more accurate and extensive study based on the RNA-sequencing approach in 27 LARC patients, detected 3 upregulated miRNAs (miR-21-5p, miR-1246 and miR-1290-3p) in complete responders (TRG 4, according to Dworak scale) to neoadjuvant therapy (5-FU plus 50.4-54 Gy) [71]. An independent validation set of patients (*n* = 16) confirmed miR-21-5p as a potential biomarker for the prediction of complete response to CRT. Appropriately, in silico prediction identified *special AT-rich sequence binding protein 1 (SATB1)* as a target gene of miR-21-5p. Moreover, functional experiments confirmed not only the interplay between miR-215p and *SATB1*, but also their direct role in response to chemoradiation [71]. The detailed information of the patients and the assessment of the tumor response at long intervals (12 weeks) from neoadjuvant chemotherapy, the validation cohort, even if small, the in vitro experimentations, but especially the use of RNA-sequencing techniques, which provides a more complete view of miRNA transcriptional landscape, are a big plus in this study compared to those previously mentioned.

miRNA-194 has been described as a potential predictor of response to nCRT through a miRNA expression profiling approach combined with integrative computational biology [72]. miRNA-194 was highly expressed in endoscopic tumor biopsies collected before nCRT and before surgery that were analyzed by microarray, and then validated by RT-qPCR and in situ hybridization (ISH). In addition, protein–protein interaction network and pathway enrichment analysis revealed that the molecular mechanism that triggers miR-194 overexpression involved the Wnt pathway via its downstream mediator tumor necrosis factor receptor-associated factor 6 (TRAF6). Moreover, the investigators suggested that the response to nCRT in which miR-194 overexpression is implicated, could be associated with the pathway of oxidative stress-induced senescence [72].

Likewise, miR-548c-5p, miR-548d-5p and miR-663a were described as promising biomarkers for predicting response to nCRT in LARC patients with pCR when compared to pathological incomplete response [73]. In particular, these miRNAs could act as complex that regulates the response to nCRT by targeting genes associated with CRC, including the gene *mutated in colorectal cancers* (*MCC*), the gene *interleukin-6 signal transducer* (*IL6ST*), and the gene *cell cycle checkpoint kinase 2* (*CHEK2*), as well as the gene *marker of proliferation Ki67* (*MKI67*) [73]. Wet-lab investigations based on the employment anti-miRNAs oligonucleotides could effectively validate the therapeutic potential of this complex to improve response to nCRT in the clinical practice.

Contrarily to prior study, recent evidence indicated that high expression levels of miR-519b-3p promoted responsiveness to nCRT in LARC patients (*n* = 55), through a microarray technique that has been validated by both RT-qPCR and ISH methods [74]. Functional investigations performed on CRC cell lines, and in vivo experimentations, revealed that this mechanism was related to the direct binding of miR-519b-3p to the 3′UTR region of its target gene, namely *AT-Rich Interaction Domain 4B* (*ARID4B*) [74].

High expression levels of miR-487a-3p were recently identified through RNA-sequencing approach in tumor tissue biopsies of non-responder patients diagnosed with LARC and treated with capecitabine or 5-FU-based nCRT [75]. This result was confirmed by RT-qPCR and by ROC curve analysis. Moreover, gene ontology (GO) and Kyoto Encyclopedia of Genes and Genomes (KEGG) pathway enrichment analyses were conducted to gain indirect insights into the biological processes that miR-487a-3p may regulate in the response to nCRT [75]. These latter in silico investigations revealed a potential connection between miR-487a-3p and the epithelial–mesenchymal transition that needs to be verified by wet-lab experimentations.

Altogether the discrepancies among the aforementioned studies indicate that miRNAs are not (yet) useful as predictive biomarker to stratify LARC patients that are effectively eligible to nCRT treatment. An interesting recent published study, in fact, reviewed the literature concerning the miRNAs identified to date as predictive biomarkers in LARC, based on a systematic research in order to summarize the data that are associated to nCRT [76]. In the screening of 61 articles, a total of 77 miRNAs were identified as holding predictive value. However, only six miRNAs (let-7f, miR-21, miR-145, miR-622, miR-630 and miR-1183) exhibit significant differences in 2 or more independent studies.

## 4. Circulating Biomarkers to Predict Response to nCRT in LARC

In the era of precision medicine, nevertheless the several advantages of liquid biopsy derived from non-invasiveness, and rapidity as well as less cost of the test, the pre-analytical variability, and particularly the low detection sensitivity limited its clinical utility in the management of LARC, which is always captained by the gold standard, the tissue biopsy.

**Figure 2:**
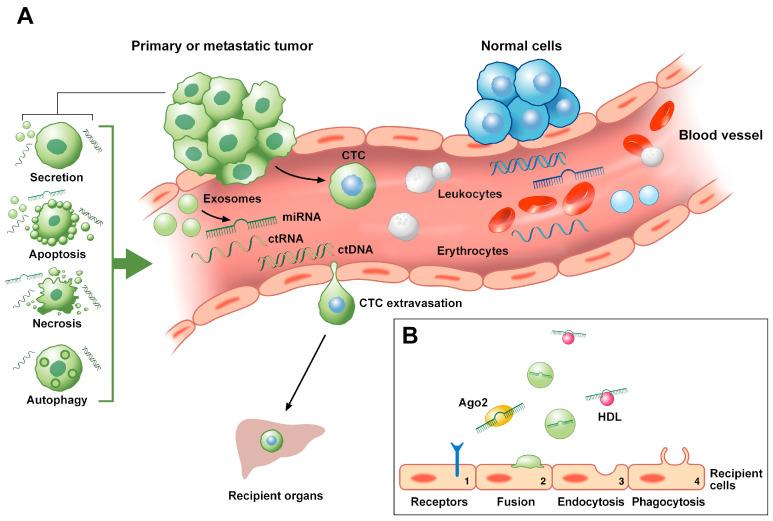
Mechanisms of release into the bloodstream and of migration to recipient cells of circulating tumor biomarkers. (**A**) CTCs usually detach from the primary or metastatic tumor and transmigrate through the vessel wall barrier to circulate into the bloodstream of LARC patients. During their travel in blood, CTCs become entrapped into microvessels. As a consequence, such travelers establish metastasis in recipient tissues either cause the rupture of the microvasculature or through extravasation. ctDNAs and ctRNAs release includes apoptosis, autophagy, necrosis, lysis of CTCs and active secretion from tumor cells. ctRNAs include extracellular vesicles-associated circulating RNA as well as a variety of RNA classes (i.e., lncRNAs, miRNAs). Among them, miRNAs can be released into the blood circulation from the lysis of tumor cells (i.e., necrosis, apoptosis) or from active secretion (i.e., exosomes or apoptotic bodies). (**B**) miRNAs floating in the blood can be present as cell-free miRNAs, being associated with RNA-binding proteins (i.e., Ago2) or lipoproteins (i.e., HDL), or be packaged inside microvesicles, such as exosomes. Circulating miRNAs are internalized by recipient cells through different mechanisms, including (1) the capture by specific cell receptors, (2) direct fusion with the plasma membrane of the receiving cells, (3) endocytosis and (4) phagocytosis. Ago2, protein argonaute 2; CTC, circulating tumor cell; ctDNA, circulating tumor DNA; ctRNA, circulating tumor RNA; HDL, high-density lipoprotein; LARC, locally advanced rectal cancer; lncRNA, long non-coding RNA; miRNA, microRNA. Circulating tumor analytes are represented in green color, while the non-cancerous ones in blue color.

Liquid biopsy consists of the non-invasive detection and analysis of the circulating components that are present in body fluids, also defined as “tumor circulome” [77]. Human bloodstream, for instance, is not only composed of different types of circulating cells, which embrace circulating tumor cells (CTCs), but can also include extracellular vesicles (i.e., exosomes), circulating derived tumor-proteins as well as a small amount of circulating cell-free nucleic acids, such circulating DNA (ctDNA) or circulating RNA (ctRNA), originating from primary and metastatic lesions (Figure 2) [77]. ctRNAs comprise extracellular vesicles-associated circulating RNA and a variety of RNA classes (i.e., mRNA, long non-coding RNAs and miRNAs). Differently from ctRNAs, to date, ctDNAs and CTCs are the only circulating analytes that are approved as clinical biomarkers by the US Food and Drug Administration (FDA) [77,78]. However, only a few studies have focused on the potential role of ctDNA and CTCs as well as circulating derived tumor-proteins in LARC.

### 4.1. Circulating microRNAs as Predictors of Responsiveness to nCRT in LARC Patients

As an alternative to the biopsy-based tissue analysis, measurements of the abundance of circulating miRNAs have been performed. miRNAs are, in fact, valid and promising non-invasive biomarkers thanks to their presence in a variety of body fluids (i.e., blood, urine and breast milk) [79]. Extracellular miRNAs can be released into the blood by different mechanisms, including active secretion by microvesicles (i.e., exosomes), apoptotic bodies, or as bounded to high-density lipoproteins (HDL), as well as ribonucleo-protein complexes (Figure 2) [80,81,82]. Concerning their mechanisms of action, evidence demonstrated that miRNAs are taken up by receiving cells through several processes, such as endocytosis, phagocytosis, and direct fusion with the plasma membrane of recipient cells (Figure 2) [83]. However, the precise mechanisms responsible of miRNAs release, as well as miRNAs internalization by recipient cells, have not been fully characterized yet [84].

Currently, only a few studies have explored the ability of circulating miRNAs as predictive biomarkers in LARC. Circulating miRNAs are listed in Table 2. For instance, plasma specimens of 42 LARC patients were explored at different time points (before, during and after CRT) to explore changes in miRNAs levels. Among five miRNAs detected (miRNA-17, 18b, 20a, 31 and 193-3p), miR-18b and miR-20a showed a decrease of the level of expression when evaluated prior and at the end of the therapy (Table 2). In addition, their reduced expression was associated with postoperative lymph node negativity. The analysis of the ROC curves validated the putative role of miR-18b and miR-20a as plasma biomarkers in predicting poor response to nCRT [85].

D’Angelo and collaborators identified miR-125b as a promising biomarker of response to nCRT (Table 2) [86]. In particular, high level of miR-125b expression in cancer tissues and serum specimens of LARC patients was associated with poor response to nCRT [86].

Similarly, low serum levels of miR-143 were associated with a pathological response to nCRT in 94 patients (Table 2) [87]. In this study, the authors, by comparing pre- and post-nCRT, demonstrated that serum levels of miR-143 after CRT were significantly lower than before CRT [87].

Through next-generation sequencing (NGS) approach, miR-100-5p and 23 miRNA variants (isomiRs) were detected differentially expressed between LARC patients who received preoperative CRT (50 Gy/25 fractions and capecitabine twice daily (825 mg/m^2^)) and patients who did not receive any treatment before serum collection (Table 2) [88]. In particular, the levels of expression of miR-100-5p and 22 isomiRs, with the exception of miR-150-5p, were significantly higher in LARC serum specimens when detected after than before CRT treatment [88].

Another study reported that low miR-345 levels extracted from serum of LARC responder patients (TRG 1–2) were associated with chemoradiation sensitivity when compared to non-responders (TRG 3–4) and with superior 3-year local recurrence-free survival (Table 2) [89].

### 4.2. Exosomal microRNAs in LARC

As described before, miRNAs could be differently released in the extracellular environment via membrane-bound vesicles, such as exosomes (Figure 2) [84]. Thereby, miRNAs are also protected from degradation [84].

An interesting prospective study of 2017 evaluated changes in the expression levels of serum miRNAs of 40 LARC patients during chemoradiation (at baseline, week 3 and at completion of chemoradiation) [90]. miR-125b-1, miR-1183 and miR-130a were downregulated 2-fold more in serum samples collected at the end of the treatment than in those collected at baseline. In addition, non-responder patients to CRT presented low expression levels of miR-130a when compared to the responders (Table 2) [90].

Recently, miR-301a-3p appeared low expressed in plasma exosome specimens of 29 patients affected from LARC with poor tumor response (TRG 2–3) to CRT (Table 2) [91]. However, the low level of significance and the failure of independent validation indicate the need of further experimentations.

A panel of highly expressed serum exosomal miRNAs (miR-21-5p, miR-1246, miR-1229-5p and miR-96-5p) has been reported to significantly identify chemoresistant LARC patients (Table 2) [92]. Finally, high expression level of the exosomal miR-199b-5p has been identified as a novel potential non-invasive biomarker to predict good response of preoperative chemoradiotherapy [93] (Table 2).

Generally, the clinical application of circulating miRNAs as biomarkers has several issues [79]. In fact, although circulating miRNAs can reflect the tumor status, at present, this relationship has to be further investigated. Moreover, even if the small size of miRNAs renders them highly stable, their quantification is difficult, especially due to their small size and also low abundance that may vary according to the type of body fluid within the same patient (i.e., usually higher in serum than in plasma specimens) [79].

At present, there are no single or panels of circulating miRNAs that can be employed in the management of LARC. Much investigation remains to be done in order to clinically introduce fluctuating detectable circulating miRNAs as a predictive biomarker of responder patients who underwent CRT.

### 4.3. Circulating Tumor DNA (ctDNA) in LARC

Studies have reported controversial results concerning the size of circulating DNA derived from neoplastic or normal tissues. It has been demonstrated that generally the main source of cell-free DNA (cfDNA) in healthy individuals is the apoptosis, which generates DNA fragments of less than 200 base pair (bp) in length [77,94]. In contrast, cancer patients have longer circulating DNA fragments that are released through necrosis or autophagy (Figure 2) [77]. Additionally, longer DNA fragments are also more abundant in the circulation of cancer individuals when compared to healthy people [77,94]. Thus, it is possible to detect a cancer by measuring the degree of the integrity of cfDNA, namely “cfDNA integrity index” [95,96,97,98]. This latter is usually calculated as a ratio between longer and shorter DNA fragments [96,97].

Currently, the most common method to detect DNA integrity is based on the measurement of the concentration of cfDNA via the quantification of *Arthrobacter luteus* (ALU) sequences by RT-qPCR [95,96,97]. ALUs are the most abundant repeated sequence in human genome of about 300 bp in length [99]. Usually, the primers used for the RT-qPCR include: (i) the primer ALU 115, which amplifies small fragments that originate from apoptosis; (ii) and the primer ALU 247 that conversely amplifies longer DNA fragments [96,97].

Furthermore, a variety of genetic (i.e., copy number aberration and single nucleotide variants) and epigenetic (i.e., status of methylation) information could be obtained from the analysis of ctDNA fractions [77]. These genetic and epigenetic information of ctDNAs usually reflect the genome and epigenome of the cell of origin [94].

Only a few investigations, and not without conflicting results, tried to demonstrate that monitoring the variation of the abundance of ctDNA and measuring the cfDNA integrity in plasma of LARC patients could be promising to predict responsiveness to nCRT [100,101,102,103]. However, the ability of nCRT in changing the amount of ctDNA has to be verified too.

Zitt and collaborators evaluated the amount of ctDNA in plasma of LARC patients undergoing preoperative CRT by using RT-qPCR before and after the end of chemoradiation, and at the end of the treatment [101]. A decrease of ctDNA in responders, while an increase of ctDNA in non-responder patients was evidenced at the end of the treatment. Thus, these results suggested that the analysis of cfDNA levels after therapy could be used to monitor radiochemotherapy efficacy [101]. Similarly, by evaluating variations of ALU 245, ALU 115, and ctDNA integrity index (ALU 247/215) in plasma specimens of 65 LARC patients before and after CRT, the post-CRT levels of both the cfDNA and of the integrity index decreased significantly in responder with respect to non-responder individuals [103].

Carpinetti and co-authors tried to assess the utility of biomarkers and liquid biopsy to tailor the management of rectal cancer patients [102]. Through the genomic study of four rectal tumors, they managed to identify patient-specific chromosomal rearrangements that were used to monitor ctDNA, DNA fragments, carrying tumor-specific genetic alterations, who are shed into the bloodstream by tumor cells. Serial blood samples were collected prospectively at diagnosis, during the resting interval, at the time of clinical evaluation of response, and during follow-up, for all of four patients, in order to detect ctDNA in the plasma of cancer patients. The levels of ctDNA dropped significantly after one week of nCRT, indicating that the absence of ctDNA was a good response to nCRT [102].

Data from another study showed the prognostic potential of the evaluation of ctDNA at the diagnosis [104]. In fact, high baseline plasma levels of total ctDNA in patients with LARC were associated with the development of local and distant recurrences, shorter DFS, as well as treatment failure, consistent at any time point during the follow-up period after surgery [104].

In a recent investigation, the analysis of ctDNA in plasma samples of 96 eligible LARC patients collected after nCRT and after surgery demonstrated that the presence of postoperative ctDNA could stratify LARC patients according to the risk of disease recurrence, independently of the nCRT [105]. Moreover, the exploitation of the ctDNA could also identify LARC patients at risk of developing metastases during the neodjuvant period and after surgery [106].

In conclusion, taking into account the published data, there is still a knowledge gap concerning the clinical utility of the pre-treatment and serial (pre-treatment and post-treatment) monitoring of ctDNA as predictive biomarker in LARC. The evaluation of the ctDNA levels able to stratify LARC patients that could benefit from nCRT and/or could be eligible for de-escalation of nCRT required to improve its predictive validity.

### 4.4. Circulating Tumor Cells (CTCs) in LARC

CTCs are circulating cancer cells that invade into the blood circulation from their primary tumors and move to distant recipient tissues where they can metastasize though either the rupture of the microvasculature or extravasation (Figure 2) [77,107]. Usually, in healthy individuals CTCs travel in the bloodstream in a small amount, which significantly increases in a pathological condition [77,108]. A minority of CTCs could also be present as clusters of CTCs that have stable cell-cell junctions [108]. These latters are associated with worse clinical outcome when compared to a single CTC [107,109]. Moreover, recent in vivo evidence showed that CTC clusters presented a stronger invasive potential over single CTCs [110].

Data from different investigations achieved concordant results on the powerful and potential predictive function of CTCs in LARC. In particular, at baseline responder patients to nCRT had a significantly higher CTC detection rate compared with non-responders. However, the CTC detection rate significantly decreased after nCRT for responders [101,111,112,113].

Recently, the role of CTCs in LARC patients undergoing nCRT followed by surgery has been explored by evaluating the predictive values of the enzyme thymidylate synthase (TYMS), the main target of 5-FU and the ultraviolet excision repair protein, RAD23 homolog B (RAD23B) before and after nCRT [114]. This study revealed that *TYMS* mRNA and/or TYMS/RAD23B protein expression in CTCs have the potential to predict non-response to nCRT and avoid unnecessary radical surgery for LARC patients with pCR [114].

### 4.5. Circulating Derived Tumor-Proteins in LARC

As described before, liquid biopsies are also based on the identification of circulating proteins for personalized treatment in cancer patients [77].

By proteomically analyzing the serum samples of 20 LARC patients who underwent to nCRT using surface-enhanced laser desorption/ionization-time of flight mass spectrometry (SELDI-TOF-MS), 14 protein peaks were identified as able to differentiate between good (TRG 1+2) and poor responders (TRG 3–5), with 87.5% sensitivity and 80% specificity [115].

More recently, novel biomarkers useful to identify patients according to nCRT response have been revealed through transcriptomic-based secretome approach. Particularly, 17 potentially secreted candidates were identified as over-expressed in LARC and associated with nCRT response [116].

The use of specific biomarkers and liquid biopsy has proven to be effective in monitoring response to treatment, in detecting disease recurrence, even before radiological or laboratory evidence. Obviously, this opens an important scenario on the personalized management of these patients: the early identification of disease progression could allow to identify a greater number of treatable metastases or could induce a change of treatment regimen; likewise, identifying negative ctDNA levels could help to avoid unnecessary surgery. Thus, the development of circulating-biomarker tests could aid the clinical decision-making in selecting LARC patients who are effectively surgical candidates or fit for a less invasive approach.

## 5. Single Nucleotide Polymorphisms (SNPs) at miRNAs in LARC

Single nucleotide polymorphisms (SNPs), as the name suggests, are caused by a single nucleotide variation in the DNA sequence [117]. Being the most common type of heritable genetic variation in humans, SNPs are used to study genetic differences between individuals and populations [117].

Many SNPs doesn’t affect gene functions, being they naturally present at DNA level [118]. However, in some cases, such sequence variations can also impact gene expression and be implicated in the development of cancer [117]. Additionally, they have been described also as potential prognostic and predictive biomarkers [119].

SNPs can occur in coding or non-coding regions of the genome, such as in miRNAs (miR-SNPs) [120]. It has been demonstrated that SNPs could be located in the miRNA sequences, in miRNA genes or in miRNAs binding sites. miR-SNPs might affect the individual’s susceptibility by causing loss or gain of miRNA functions, or by altering the epigenetic regulation of miRNA encoding genes, or again by affecting the pri- and pre-miRNA processing or the interaction between miRNAs and their target mRNAs [120].

Few studies focused on the involvement of miR-SNPs in LARC [121,122,123]. For instance, the prognostic role of the SNP rs4919510, that is characterized by the guanine to cytosine (G > C) base substitution in the sequence of the mature miRNA-608, has been explored in LARC patients subjected to neodjuvant capecitabine and oxaliplatin (CAPOX) treatment followed by CRT, surgery, or to adjuvant CAPOX ± cetuximab treatment [121]. This retrospective study showed that the CC genotype was associated with a worse 5-year progression-free survival (PFS) in LARC patients treated with chemotherapy with respect to the CG/GG genotype patients. Furthermore, the administration of cetuximab to the chemotherapy and CRT, significantly improves the 5-year PFS and OS in CC carriers when compared to CG/GG genotypes [121]. This study evidenced not only the role of miR-SNPs in the risk of cancer, but also their impact in drug response.

The rs61764370 SNP (thymine to guanine (T > G) base substitution) has been identified as potential biomarker of response to neoadjuvant treatment and of a favorable outcome for LARC patients [122]. This polymorphism, harbored in the complementary site 6 (LCS-6) of the tumor suppressor miRNA let-7, alters the affinity between let-7 and its target oncogene KRAS, and consequently increasing cancer proliferation [122]. In particular, it has been demonstrated that LARC individuals with LCS-6 TG genotype reached complete response after neoadjuvant treatment and showed a better 5-years PFS and OS when compared to the TT genotype group [122].

In another study, 265 Caucasian LARC patients (divided in two subgroups depending on the radiation dose) subjected to neoadjuvant CRT based on 5-FU were screened for a panel of 114 miR-SNPs [123]. A total of five SNPs were identified in miRNAs target genes. In detail, two SNPs in SMAD Family Member 3 (SMAD3) (rs744910 and rs745103) and one SNP in transactivation response element RNA-binding protein (TRBP) (rs6088619) were predictive of pCR. In contrast, the SNPs rs10719 (in Drosha) and rs17228212 (in *SMAD3)* had an unfavorable chance of pCR [123].

These studies highlight the great potentialities of the SNPs as novel guide in response prediction to nCRT in LARC. However, further studies and validations will strengthen the clinical importance and use of these latters in stratifying LARC patients according to their genotype in order to identify the most appropriate treatment approach.

## 6. Conclusions

Interlinked disciplines collaborated to improve the management of RC that, in the past years, has been dominated by the dramatic surgery approach. At present, neoadjuvant chemoradiation in LARC care has the potential to reduce the tumor size for an effective oncological resection and the local risk of recurrence. Unfortunately, the responsiveness to nCRT has a wide range of variability. Thus, the assessment of the variation of levels of single or signatures of miRNAs in tumor specimens or, even better circulating miRNAs and/or tumor cells in body fluids, could improve the stratification of LARC patients according to the nCRT response, consequently facilitating the clinical decision-making.

Alterations in the abundance of miRNAs and circulating tumor markers are involved in the pathogenesis of various types of human cancers, including LARC. Moreover, they show a great potential as non-invasive biomarkers; miRNAs, for instance, due to their stability as tumor-derived cell-free molecules. Their clinical relevance in human diseases as diagnostic, prognostic, and therapeutic biomarkers is also evidenced by the number of clinical trials that at present have been completed (clinicaltrials.gov). However, only a few potential biomarkers are currently used in the clinical practice [124,125,126,127,128]. To date, the European medicines agency (EMA) and FDA approved the detection of mutations of the *EGFR* gene from ctDNA in order to select patients affected by non-small cell lung cancer who are eligible for treatment with erlotinib (FDA), afatinib (FDA), gefitinib (EMA), or osimertinib (EMA and FDA), thus avoiding biopsies for some patients [124,125,126]. The FDA has also approved a blood-based test for the early detection of CRC patients, namely the EpiproColon^®^ test [127]. This latter is a qualitative assay based on the ctDNA analysis for the detection of methylated Septin9 DNA, whose hypermethylated status is associated with CRC [127]. Concerning CTCs analysis, the CellSearch^®^ test is the only one that has been approved by the FDA for the prognosis of CRC, breast and prostate tumors [128].

In this review, we have first reported the most relevant studies that have occurred over the years on the identification of altered miRNAs, whose deregulated expression could be predictive of nCRT responsiveness.

Several investigators have attempted to profile aberrant miRNAs in the complete pathological response to neoadjuvant therapy in LARC patients. Despite the interesting biological information derived from the different data, at present, no reliable miRNA has been identified yet that can effectively predict the response to nCRT. In fact, looking at the different published studies, discrepancies clearly appeared in the reported levels of expression of the miRNAs in responder and no responder LARC patients. The biological variability (heterogeneity of tumor biopsies), as well as the pre-analytical (type of samples, method of collection, storage and of sample processing) and post-analytical variability, and again the different techniques applied for the detection of miRNAs have clearly influenced the comparability of the results. Moreover, the clinical applicability of the miRNAs identified still lack a cohort and different techniques of validation, as well as functional investigations on the intrinsic mechanism that regulate their altered expression and their targets.

Then, we have also discussed the attractive strategy in identifying changes in the tumor response to nCRT based on the minimally invasive liquid biopsy by exploring the relevant and recently published investigations on circulating miRNA and tumor cells, as well as circulating-free nucleic acids.

The “tumor circulome” screening could have the potential to effectively change the medical approach to the management of LARC care. Data from different studies clearly indicate that the levels of CTCs after nCRT significantly decrease in responder patients. However, there are still numerous questions regarding the predictive role of these circulating analytes, as well as extensive massive validations that deserve future investigations.

In summary, it still remains to be determined whether LARC patients can be advantageously stratified based on the levels of expression of miRNAs and other circulating tumor markers. We envision that this gap of knowledge will be filled by future studies that will use next-generation technologies, ultimately improving the quality of life and therapeutic outcome of rectal cancer patients.

## Figures and Tables

**Table 1:** The Most Commonly Used Tumor Regression Grade (TRG) Systems.

Scale	TRG	Description
Mandard	1	complete regression, no viable cancer cells, fibrosis extending through the different layers of the esophageal wall
	2	rare residual cancer cells scattered through the fibrosis
	3	increased number of residual cancer cells, fibrosis predominates
	4	residual cancer outgrowing fibrosis
	5	absence of regressive changes
Dworak	0	no regression
	1	minimal response, dominant tumor mass, fibrosis and/or vasculopathy
	2	moderate response, dominant fibrotic changes and a few easy-to-find tumor cells or groups
	3	near-complete response with few microscopically difficult-to-find tumor cells in fibrotic tissue with or without mucous substance
	4	complete response, no tumor cells and only fibrotic mass or acellular mucin pools
Ryan	0	complete response, no viable cancer cells
	1	near-complete response, single cells or rare small group of cancer cells
	2	partial response, residual cancer with evident tumor regression, but more than single cells or rare small groups of cancer cells
	3	poor or no response, extensive residual cancer with no evident tumor regression

TRG, tumor regression grade.

**Table 2:** Circulating miRNAs Associated with Response to Neoadjuvant Chemotherapy in Locally Advanced Colorectal Cancer.

miRNA	Biological Source	Level of Expression to Predict Good Response to nCRT
miR-18b and miR-20a	plasma	high
miR-125b	serum	low
miR-143	serum	low
miR-100-5p	serum	low
miR-345	serum	low
miR-125b-1, miR-1183 and miR-130a	serum	high
miR-301a-3p	plasma exosomes	high
miR-21-5p, miR-1246, miR-1229-5p and miR-96-5p	serum exosomes	low
miR-199b-5p	serum exosomes	high

miRNA, microRNA; nCRT, neoadjuvant chemoradiotherapy.

## Supplementary Materials

The following are available online at https://www.mdpi.com/1422-0067/21/19/7040/s1, Supplementary Table S1. microRNAs emerged as predictive tissue biomarkers of response to neoadjuvant chemoradiotherapy in locally advanced rectal cancer.

## Abbreviations

5-FU	5-Fluorouracil
Ago2	Argonaute2 protein
AJCC	american joint committee on cancer
ALU	Arthrobacter luteus
ARID4B	AT-Rich Interaction Domain 4B
bp	base pair
c-Myc	Avian myelocytomatosis virus oncogene cellular homolog
CAPOX	capecitabine and oxaliplatin
cCR	complete clinical response
cf	cell-free
cfDNA	cell-free DNA
CHEK2	cell cycle checkpoint kinase 2
CI	confidence interval
CIMP+	CpG island methylator phenotype positive
CLE	confocal laser endomicroscopy
CNVs	copy number variations
CpG	C phosphate G
CRC	colorectal cancer
cT	tumor stage
CT	computed tomography
CTC	circulating tumor cell
ctDNA	circulating DNA
ctRNA	circulating RNA
DE	differentially expressed
DFS	disease-free survival
DNA	Deoxyribonucleic acid
DRe	digital rectal examination
EGFR	epidermal growth factor receptor
EMA	european medicines agency
FDA	US food and drug administration
FFPE	formalin-fixed, paraffin-embedded
G>C	guanine to cytosine
GO	gene ontology
Gy	gray
HDL	high-density lipoprotein
HR	hazard ratio
IGFR	insulin-like growth factor receptor
IL6ST	interleukin-6 signal transducer
ISH	in situ hybridisation
isomiR	miRNA isoform
IWWD	international watch & wait database
kb	kilobase
KEGG	kyoto encyclopedia of genes and genomes
KRAS2	kirsten rat sarcoma viral oncogene 2
LARC	locally advanced rectal cancer
LCS-6	Let-7 complementary site 6
let-7	lethal-7
LNA	locked nucleic acid
lncRNA	long non-codign RNA
MCC	mutated in colorectal cancers
miR-SNP	microRNA-related single nucleotide polymorphism
MIR17HG	miR-17-92a-1 cluster host gene
miRNA/miR	microRNA
MKI67	marker of proliferation Ki67
MRI	magnetic resonance imaging
mRNAs	messenger RNA transcripts
MSI+	microsatellite instability positive
MV	megavolt
N+	metastatic perirectal lymph-nodes
nCRT	neoadjuvant chemoradiation
NGS	next-generation sequencing
oncomiR	oncogenic miRNA
OnCoRe	oncological outcomes after clinical complete response in patients with rectal cancer
OS	overall survival
PARP3	poly (ADP-ribose) polymerase 3
pCR	complete pathological response
PET/CT	positron emission tomography/computed tomography
PFS	progression-free survival
RAD23B	RAD23 homolog B
RC	rectal cancer
RCT	randomised controlled trials
RECIST	response evaluation criteria in solid tumors
RNA	Ribonucleic acid
ROC	receiver operating characteristic
RT	radiotherapy
RT-qPCR	realtime- quantitative polymerase chain reaction
SATB1	special AT-rich sequence binding protein 1
SELDI-TOF-MS	surface-enhanced laser desorption/ionization-time of flight mass spectrometry
SMAD3	SMAD Family Member 3
SNP	single nucleotide polymorphism
SUV	standard uptake value
T	tesla
T classification	tumor classification
T>G	thymine to guanine
TaTME	transanal surgery
TME	total mesorectal excision
TNM system	tumor, node, methastasis system
TP53	tumor protein 53
TRAF6	tumor necrosis factor receptor-associated factor 6
TRBP	transactivation response element RNA-binding protein
TRG	tumour regression grade
TRUS	transrectal ultrasonography
TYMS	thymidylate synthase
UTRs	3′-untranslated regions
vs	versus
Wnt	Wingless-related integration site
